# Separation and Enrichment of Alkaloids from Coptidis Rhizoma and Euodiae Fructus by Macroporous Resin and Evaluation of the Effect on Bile Reflux Gastritis Rats

**DOI:** 10.3390/molecules27030724

**Published:** 2022-01-22

**Authors:** Yan-Ying Li, Jin-Lei Feng, Zheng Li, Xin-Yu Zang, Xiu-Wei Yang

**Affiliations:** 1 State Key Laboratory of Natural and Biomimetic Drugs, Department of Natural Medicines, School of Pharmaceutical Sciences, Peking University Health Science Center, Peking University, Beijing 101191, China; yanyingli@ykrskj.com; 2 TCM R&D Center, Beijing Increase Pharm Co., Ltd., Beijing 102200, China; 3 TCM R&D Center, Tianjin Increase Innovative Drug Co., Ltd., Tianjin 300382, China; fengjinlei@ykrskj.com (J.-L.F.); lizheng@ykrskj.com (Z.L.); 4 Academy for Advanced Interdisciplinary Studies, Peking University, Beijing 100191, China

**Keywords:** bile reflux gastritis, Coptidis Rhizoma, Euodiae Fructus, enrichment and purification, gastric pathology, macroporous resin, rats, total alkaloids, UHPLC–ESI–QTOF-MS, UHPLC–DAD

## Abstract

The Zuojin Pill consists of Coptidis Rhizoma (CR) and Euodiae Fructus (EF). It has been a classic prescription for the treatment of gastrointestinal diseases in China since ancient times. Alkaloids are considered to be its main pharmacologically active substances. The authors of the present study investigated the feasibility of preparing high purity total alkaloids (TAs) from CR and EF extracts separately and evaluated the effect for the treatment of bile reflux gastritis (BRG). *Coptis chinensis* Franch. and *Evodia rutaecarpa* (Juss.) Benth. were used in the study. An optimized method for the enrichment and purification of TAs with macroporous resin was established. Furthermore, qualitative analysis by using ultra-high performance liquid chromatography coupled with electrospray ionization and quadrupole-time of flight mass spectrometry (UHPLC–ESI–QTOF-MS) was explored to identify the components of purified TAs. Thirty-one compounds, thirty alkaloids and one phenolic compound, were identified or tentatively assigned by comparison with reference standards or literature data. A method of ultra-high performance liquid chromatography coupled with diode array detector (UHPLC–DAD) for quantitative analysis was also developed. The contents of nine alkaloids were determined. Moreover, a rat model of BRG was used to investigate the therapeutic effect of the combination of purified TAs from CR and EF. Gastric pathologic examination suggested that the alkaloids’ combination could markedly attenuate the pathological changes of gastric mucosa.

## 1. Introduction

**Figure 1:**
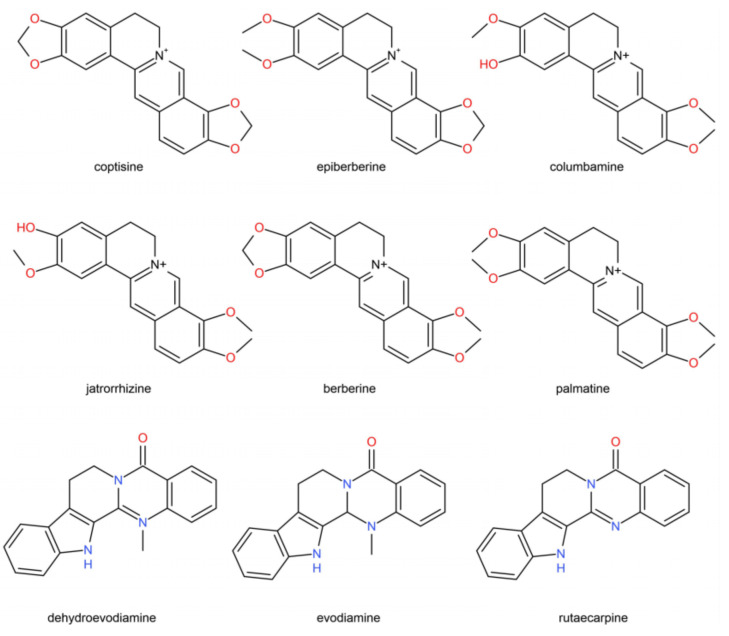
Chemical structures of protoberberine-type and indole–quinolone alkaloids.

The Zuojin Pill is a traditional Chinese medical formula. It was first recorded in Danxi’s Experiential Therapy for treating gastrointestinal disorders in the fifteenth century. This classical prescription has been used by numerous herbalists for hundreds of years. It is officially listed in the Chinese Pharmacopoeia as a prescription for patients suffering from gastric ulcer, gastroesophageal reflux disease, gastritis, and pyloric obstruction, among other disorders [1]. It consists of two commonly used herbs, namely Coptidis Rhizoma (CR, the rhizome of *Coptis chinesis* Franch.) and Euodiae Fructus (EF, the unripe fruit of *Euodia rutaecarpa* (Juss.) Benth.), with a specific dose ratio of 6:1 (*w/w*). The employment of CR and EF are contrary but complementary: Coptidis Rhizoma, the herb with bitter flavor and cold property, enters into the channels of the heart, liver, stomach, and large intestine, where it functions in clearing heat, drying dampness, purging fire, and detoxifying. Euodiae Fructus, the herb with spicy flavor and warm property, enters into the channels of the liver, spleen, stomach, and kidney, and it functions in dispersing cold and relieving pain, hindering the reversed Qi, stopping vomiting, and strengthening yang to relieve diarrhea. The results of pharmacological research have shown that the bioactivities of the Zuojin Pill are attributed to alkaloids from the herbal pair of CR and EF [2]. The primary active ingredients in CR are supposed to be protoberberine-type alkaloids, such as columbamine, and epiberberine. Indole–quinoline and –quinolone alkaloids are supposed to be the primary active components of EF [3,4] (Figure 1).

**Figure 2:**
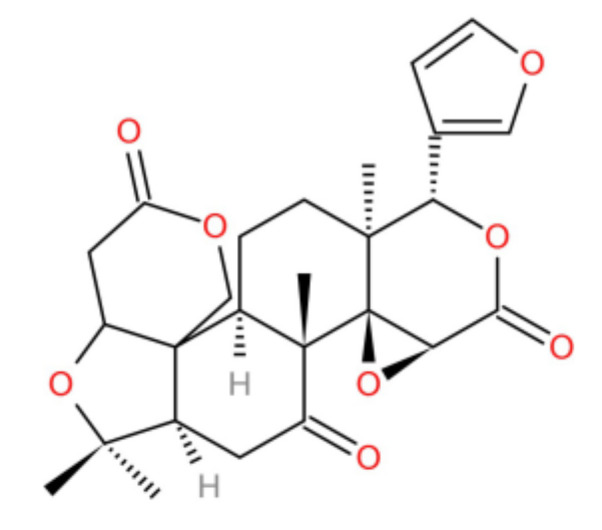
Chemical structure of limonin.

Limonin (Figure 2) belongs to the tetracyclic nor-triterpenoids in EF. At present, the pharmacological effects of limonin are known to include anti-tumor, anti-inflammatory, analgesic, anti-bacterial, anti-virus, anti-oxidation, nerve protection, liver protection, and blood lipid regulation properties. However, in recent years, the toxicity of limonin has also been reported. Some studies have shown that limonin has hepatorenal and genetic toxicity [5]. Limonin (50–200 µg·mL^−1^) can significantly inhibit the viability of human embryonic kidney cells (HEK-293 cells) in a dose-dependent manner, and it can shrink, reduce, and even kill kidney cells in varying degrees at a concentration of 100–200 µg·mL^−1^ [6]. Recent studies have shown that limonin produces various chromosomal aberrations in hamster lung cells (CHL cells) such as chromosome bridges, chromosome exchange, dicentric, circular chromosomes, and pulverization in the case of 0.025–2.5 mg·mL^−1^, as well as that the inhibition rate of limonin on CHL cells is linear. Therefore, it was believed that limonin could cause chromosome distortion in CHL cells and produce genotoxicity [7]. Further research confirmed that the main type of chromosome aberration in CHL cells by limonin is dicentric [8].

In our previous studies [9], 65 compounds, mainly including alkaloids, phenolic compounds, and limonoids, in the CR–EF herbal pair system were identified by UHPLC–ESI–QTOF-MS analysis, and an optimized UHPLC–DAD method was established for the chemical fingerprint analysis of the CR–EF herbal pair extracts and the quantitative determination of nine major alkaloids. We conducted a comprehensive study in the quality control of CR–EF herbal pairs. Upon further investigation, alkaloids from CR and EF were found to exert anti-inflammatory bioactivities. For example, berberine was reported to ameliorate lipopolysaccharide-induced inflammatory responses [10]. Palmatine has been proven as an anti-inflammatory drug for the clinical treatment of gastrointestinal infections, surgical infections, and gynecological inflammation [11]. Evodiamine, dehydroevodiamine, and rutaecarpine, the major bioactive alkaloids in EF, have also been found to have anti-inflammatory effects [12,13,14]. Alkaloids from *C. chinensis* Franch. and *E. rutaecarp*a (Juss.) Benth. could exert anti-inflammatory effects through the inhibition of iNOS, COX-2, IL-6, IL-1β, and TNF-α expression by preventing the nuclear translocation of the NF-κB p50 and p65 subunits in RAW 264.7 cells [15]. These findings provided evidence to understand the therapeutic effects of the Zuojin Pill on gastritis, gastric ulcer, and other inflammatory diseases in clinics. 

Bile reflux gastritis (BRG) is a type of chronic gastritis that accounts for about 22.6% of chronic gastritis [16]. BRG refers to inflammatory lesions of the gastric mucosa caused by the reflux of bile into the stomach [17]. Previous studies have shown that the Zuojin Pill has excellent clinical effects in the treatment of BRG [18]. However, the protective effect of the alkaloids from the Zuojin Pill on gastritis, especially on BRG, has not been clarified in vivo. 

Additionally, the preparation of TAs from the Zuojin Pill always starts with the CR–EF herbal pair, and the components from the two herbs are mixed together in a gradient elution process during macroporous resin purification, which leads to a relatively low yield of alkaloids: the quantities of two alkaloids in crude drugs of the Zuojin Pill are 4.21% berberine and 0.12% evodiamine. After purification, the contents of berberine and evodiamine are 12.93% and 0.37%, respectively [19]. Considering that limonin possesses certain genotoxicity and mutagenicity, it must be removed in the purification process. As alkaloids from EF have lower polarity than limonin, limonin can be easily removed in the gradient elution program of EF.

In this study, 70% ethanol extracts of CR and EF were separately loaded into resin columns, and an optimized method for the enrichment of TAs with macroporous resin was established. A method of UHPLC–ESI–QTOF-MS for qualitative analysis was also developed in the present study. Furthermore, we optimized a new UHPLC–DAD method to determine the major alkaloids in crude drugs and purified the TAs of CR and EF. Finally, the purified TAs of CR and EF were mixed in a certain proportion as an alkaloid combination, and this combination’s effect on BRG rats was studied. 

## 2. Results

### 2.1. Optimization of Macroporous Resin Purification of Total Alkaloids (TAs)

In order to comprehensively assess the enrichment and purification process, methods for determining the total alkaloids (TAs) of CR and EF with ultraviolet spectrophotometry were developed, as described in Section 3.5. All the following adsorption and desorption capacity values are expressed as TA content (mg TAs·g*^−^*^1^ dry resin). All values represent the mean ± SD of the three independent experiments.

**Figure 3:**
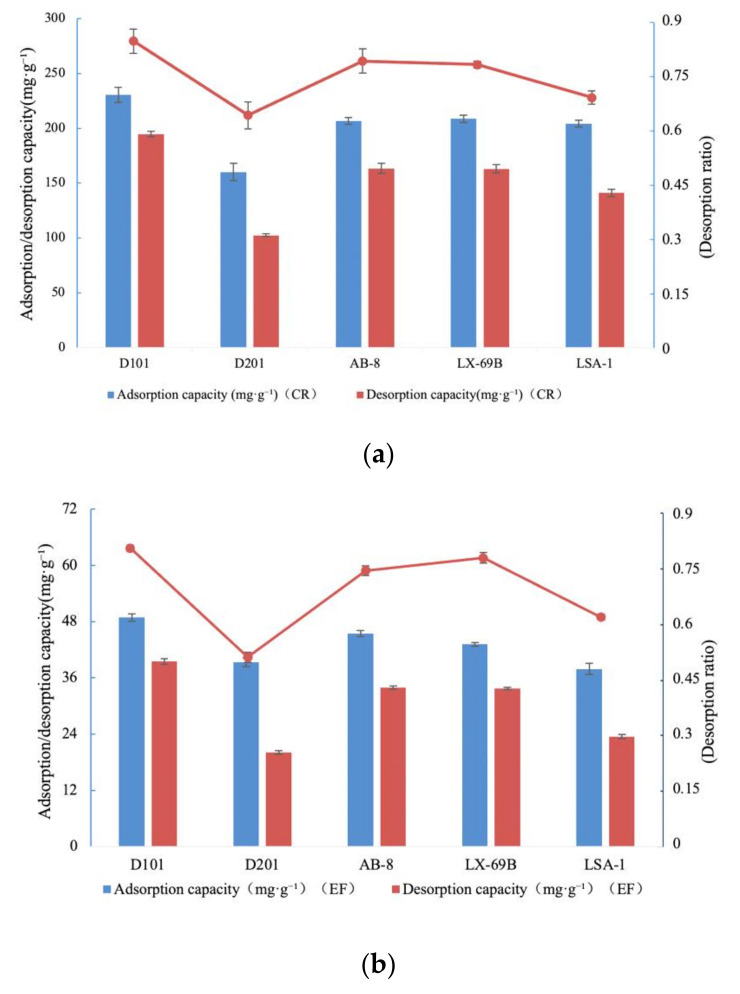
Adsorption capacities, desorption capacities, and desorption ratios of TAs from CR (**a**) and EF (**b**).

According to the “like dissolve like” rule, either non-polar resins or polar resins can be applied to the adsorption of alkaloids. Hence, five macroporous resins (D101, D201, AB-8, LX-69B, and LSA-1) were selected. As depicted in Figure 3a, the adsorption capacity (230.7 ± 6.8 mg·g^−1^ for CR, as shown in the blue bars), the desorption capacity (194.8 ± 2.5 mg·g^−1^ for CR, as shown in the red bars), and the desorption ratio (84.5 ± 3.4% for CR, as shown in the red line above) of the D101 resin were higher than those of the other resins in values. The adsorption capacity (48.9 ± 0.8 mg·g^−1^ for EF) of the D101 resin was higher than the others, and the D101 resin was finally selected for its highest desorption ratio (80.8 ± 0.9% for EF); see Figure 3b. Therefore, static adsorption and desorption experiments were further executed with the D101 resin. The physical properties of five macroporous resins are listed in Table 1.

**Figure 4:**
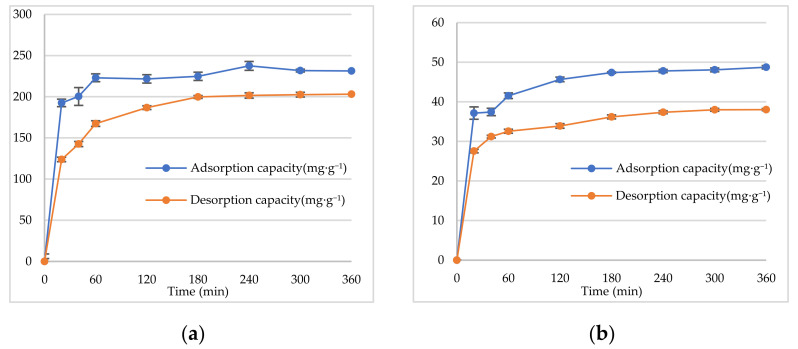
Kinetics for static adsorption and desorption capacities of the D101 macroporous resin for CR (**a**) and EF (**b**).

In the present study, the adsorption kinetics of TAs were determined to understand the adsorption behavior of the D101 resin. Static adsorption and desorption experiments were conducted at 25 °C. Figure 4a,b shows the kinetics for static adsorption and desorption capacities of the D101 macroporous resin for CR and EF, respectively. In Figure 4a, as shown by the adsorption curve, the TAs of CR were rapidly and efficiently adsorbed by the D101 macroporous resin with an adsorption equilibrium after 60 min. The adsorption capacity at 60 min was 237.50 ± 4.94 mg·g^−1^. The desorption capacity sharply increased in the first 60 min and then reached an equilibrium at 180 min. As shown in Figure 4b, the adsorption capacity of the D101 resin rapidly increased during the first 60 min and then reached an adsorption equilibrium at 180 min. After 180 min, the adsorption level did not show any further significant changes, which suggested that the adsorption equilibrium occurred at 180 min, when the adsorption capacity was 47.78 ± 0.40 mg·g^−1^. The desorption capacity sharply increased in the first 60 min and reached an equilibrium at 240 min.

**Figure 5:**
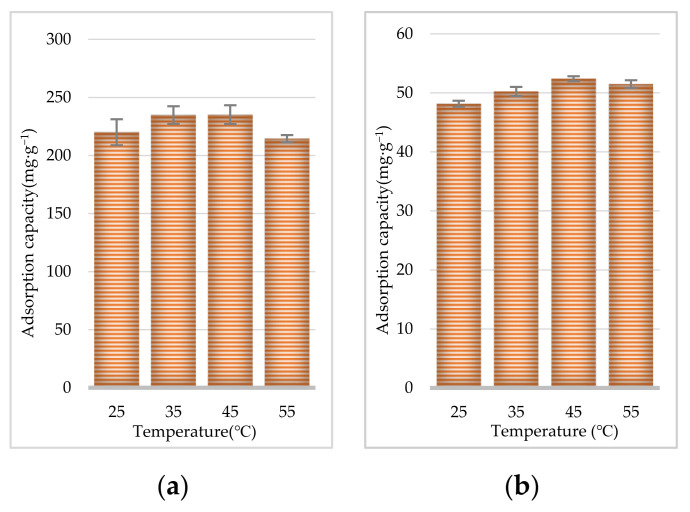
Effect of temperature on adsorption capacities of D101 resin for CR (**a**) and EF (**b**).

To investigate the adsorption capacity and characterize the adsorption behavior of CR and EF alkaloids, sample solutions of CR or EF extracts with a concentration of 0.5 g·mL^−1^ (calculated as the weight of crude drug) were shaken with the D101 resin at 25, 35, 45, and 55 °C. As shown in Figure 5a, for CR, increasing the adsorption temperature to 35 °C resulted in a slight but not significant increase in the adsorption capacity. As shown in Figure 5b, for EF, when the temperature increased, the adsorption capacity reached a maximum value at 45 °C.

**Figure 6:**
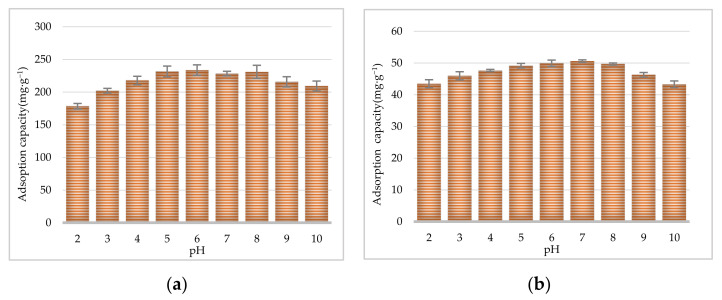
Effect of pH on adsorption capacities of D101 resin for CR (**a**) and EF (**b**).

A key parameter affecting the adsorption and desorption capacities of resins is the pH value of the initial sample solution. The pH value influences the extent of ionization of solute molecules and therefore affects the affinity between the solutes and solutions. As shown in Figure 6a for CR, with the D101 resin, the adsorption capacity of the TAs reached its crest value at pH 6.0 and then gradually decreased with the rise in pH value. As shown in Figure 6b for EF, the pH of the EF extract influenced this purification step, with an optimal obtained pH value of 7.0. 

**Figure 7:**
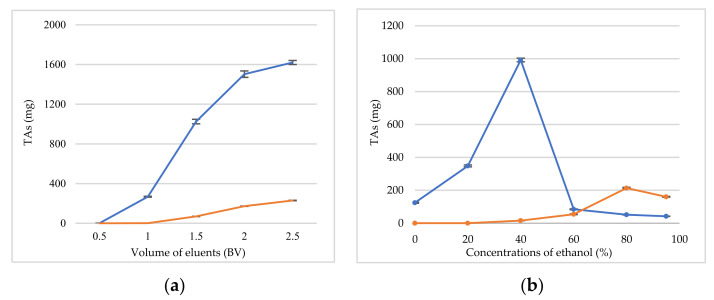
Dynamic breakthrough curve (**a**); the effect of ethanol concentrations (**b**) for CR (blue lines) and EF (yellow lines).

The dynamic leakage curve was investigated for getting the appropriate loading volume of the CR and EF extracts. Sample solutions of CR or EF extracts with a concentration of 0.5 g (calculated as the weight of crude drug)/mL were loaded onto D101 resin columns and flowed through the resin columns at the speed of 1 BV·h*^−^*^1^. As shown in Figure 7a, when the loading volume was 0.5 BV, the leakage point was observed (blue line of CR). Hence, 0.5 BV was considered to be the appropriate loading volume for CR, and 1 BV was selected as loading volume of EF (yellow line of EF). As shown in Figure 7b, the desorption of the TAs was the highest when the ethanol concentration reached 40% (blue line of CR), and 40% ethanol could desorb the great majority of alkaloids. Therefore, 40% ethanol was considered to be the best desorb solvent to elute alkaloids of CR. For EF (yellow line of EF), 60% ethanol (8 BV) was firstly used to eliminate limonin, and then 95% ethanol was able to desorb the great majority of the alkaloids. 

Thus, the ultimate separation and purification methodology for TAs can be summarized as follows. For CR adsorption: a sample solution concentration of a 0.5 g·mL^−1^ (calculated as the weight of crude drug), a loading volume of 0.5 BV, a pH of 6.0, a flow rate of 1 BV·h^−1^, and a temperature of 35 °C. For desorption: 40% ethanol to enrich the TAs of CR. For EF adsorption: a sample solution concentration of 0.5 g·mL^−1^(calculated as the weight of crude drug), a loading volume of 1 BV, a pH of 7.0, a flow rate of 1 BV·h^−1^, and a temperature of 45 °C. For desorption: 60% ethanol (8 BV) to remove limonin and 95% ethanol to enrich the TAs of EF.

### 2.2. Analysis of Alkaloids by UHPLC–ESI–QTOF-MS

**Figure 8:**
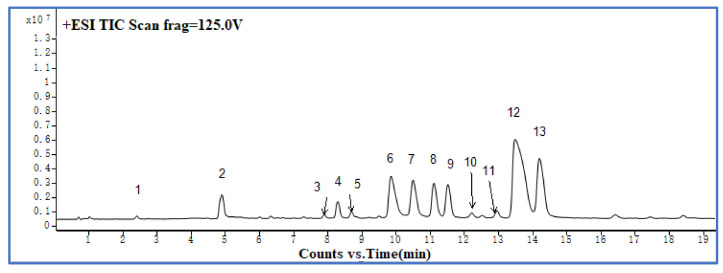
TIC profile in positive ion mode of TAs from CR prepared with D101 macroporous resin purification.

To characterize the chemical constituents in the purified TAs of CR, an UHPLC–ESI–QTOF-MS method was established. Figure 8 shows the total ion chromatogram (TIC) in positive ion mode recorded by UHPLC–ESI–QTOF-MS of the TAs of the prepared CR, under optimum conditions, with the D101 macroporous resin purification steps. As can be seen in Figure 8 and Table 2, a total of 13 compounds were identified or tentatively characterized: 12 alkaloids and 1 phenolic compound. Compounds 4, 6, 8, 9, 12, and 13 were identified to be epiberberine, coptisine, columbamine, jatrorrhizine, berberine, and palmatine through comparison with reference standards. The structures of the other seven compounds were tentatively characterized by comparing their characteristic high-resolution mass data with data from previous publications [20,21]. The mass error for molecular ions of all identified compounds was within ±5 ppm.

**Figure 9:**
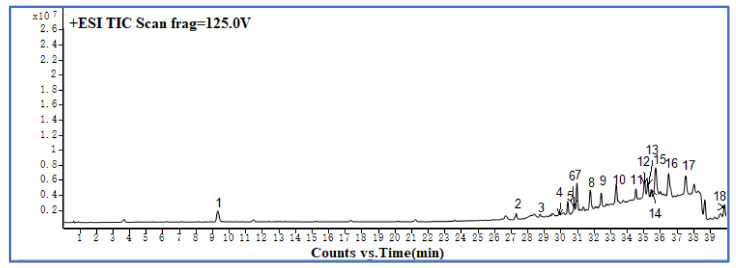
TIC profile in positive ion mode of TAs from EF prepared with D101 macroporous resin purification.

Figure 9 shows the TIC profile in positive ion mode recorded by UHPLC–ESI–QTOF-MS of the TAs of the prepared EF under optimum conditions, with the D101 macroporous resin purification steps. As can be seen in Table 3, a total of 18 alkaloids were identified or tentatively characterized. Compounds 1, 7, and 8, were unambiguously identified as dehydroevodiamine, evodiamine, and rutaecarpine, respectively, by comparing their retention time and MS data with those of reference standards. Another 15 compounds of quinolone alkaloids were tentatively assigned based on their empirical molecular formulae and literature reports [22,23,24,25,26,27,28,29]. Taking compound 18 as an example, it exhibited [M + H]^+^ ion at *m/z* 370.3099 in the positive ion mode, indicating an MW of 369. In the MS/MS spectrum, a series of characteristic ions—*m/z* 159.0676 ([C_10_H_9_NO]^+^), 173.0838 ([C_10_H_9_NO + CH_2_]^+^), and 186.0918 ([C_10_H_9_NO + C_2_H_3_]^+^)—were yielded by the fragmentation of different positions at the side chain, as described in our previous study [9]. In addition, the fragment ion at *m/z* 200.1069 was generated by the neutral loss of C_6_H_12_ from the parent nucleus. Therefore, compound 18 was tentatively identified as 1-methyl-2-pentadecyl-4(1H)-quinolone, as previously reported for this plant [29].

### 2.3. UHPLC–DAD Quantitative Analysis

Quantitative determinations of the nine compounds in Figure 1 were performed with UHPLC–DAD.

#### 2.3.1. Linearity

External standard curves covering the concentration range of each compound were established at six data points based on the estimated range of extract content, and the curves were established by the peak areas and corresponding concentration of each analyte. As shown in Table 4, the R^2^ values of the nine standard substances were all greater than 0.999, and the standard curve and linear range were determined.

#### 2.3.2. Precision, Repeatability, and Stability 

Intra-day and inter-day variations were used to characterize the measurement precision of the developed method. For the intra-day variability test, a sample solution, prepared as described in Section 3.3 was analyzed for six replicates within one day, while for the inter-day variability test, the sample was tested in duplicate for three consecutive days. To confirm the repeatability, six replicates of the same sample were extracted and analyzed, as described in Section 3.3. A sample solution was examined at 0, 2, 4, 8, 12, and 36 h to evaluate sample stability. Relative standard deviations (RSDs) of intra-day and inter-day precision were ≤2.0%, and those of stability were ≤3.0% (Table 5).

#### 2.3.3. Recovery

The accuracy of the quantification method was validated with a standard spiking test. The proposed method was applied to the samples spiked with the mixed standard solution at a 100% concentration level. Six replicate experiments at each level were performed. The ratio of detected and added amounts was used to calculate the recovery. The average recoveries of the nine analytes ranged from 97.42% to 100.80%, with all RSDs of ≤3.0%, suggesting that the method was accurate (Table 5).

#### 2.3.4. UHPLC–DAD Quantitative Analysis of Purified TAs 

Crude drugs (2 kg) of CR and EF were extracted and purified according to the optimized purification methods. The contents of the nine index components in crude drugs and purified TAs were determined with the UHPLC method. The applied chromatographic condition used for the separation of the nine compounds presented good separation. Six alkaloids comprised the CR crude drug: coptisine, epiberberine, columbamine, jatrorrhizine, palmatine, and berberine at 1.5%, 0.89%, 0.47%, 0.35%, 1.19%, and 4.83%, respectively. In the purified TAs of CR, the contents of the six alkaloids obviously increased to 9.77%, 5.41%, 3.14%, 2.24%, 7.96%, and 31.46%, respectively. In addition to CR, the contents of three major marker alkaloids in the purified TAs of EF were also significantly increased: the contents of evodiamine, rutaecarpine, and dehydroevodiamine in the EF crude drug were 0.55%, 0.28%, and 0.86%, respectively. After purification, the contents were 5.89%, 5.07%, and 0.92%. Limonin was not detected in the purified TAs of EF.

### 2.4. Inhibitory Effect of CR and EF Alkaloids’ Combination on BRG

**Figure 10:**
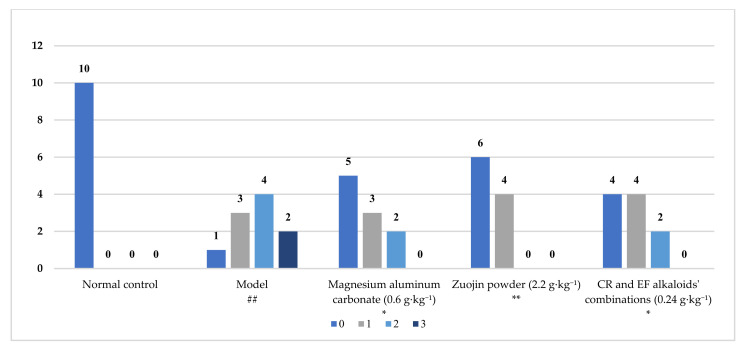
Pathological degree of gastric mucosa in reflux gastritis rats (*n* = 10). ## *p* < 0.01 vs. normal control, * *p* < 0.05 vs. model, ** *p* < 0.01 vs. model.

A rat model of BRG was used in the study. After the rat model was established, the rats were divided into five groups and treated with different procedures, as described in Section 3.9.2. The tissue samples were obtained after two weeks. The changes of the gastric mucosa were studied with microscopy. As shown in Figure 10, the pathological degree of the 10 rats in the normal control group were classified as level 0 according to the standard described in Section 3.9.4: no obvious inflammatory cell infiltration and intestinal metaplasia in gastric mucosa. In the model group, there was one rat in level 0, three rats in level 1, four rats in level 2, and two rats in level 3. The gastric mucosa of some rats in the model control group was infiltrated with inflammatory cells or intestinal metaplasia, and the infiltrated inflammatory cells were mainly mononuclear lymphocytes. Compared to the normal control group, the pathological changes in the model group showed significant differences (*p* < 0.01). In the magnesium aluminum carbonate group, there were five rats in level 0, three rats in level 1, and two rats in level 2. In the Zuojin powder group, there were six rats in level 0, four rats in level 1, and no rat in level 2 or 3. In the alkaloids′ combination group, there were four rats in level 0, four rats in level 1, and two rats in level 2. Inflammatory cell infiltration or intestinal metaplasia in gastric mucosa was observed in the magnesium aluminum carbonate group and two other therapy groups, and the pathological changes showed significantly differences compared with the model group (*p* < 0.05 or *p* < 0.01). Compared to the Zuojin powder group, the alkaloids′ combination group exhibited a little weaker therapeutic potential in BRG rats at a dose of 0.24 g·kg^−1^. 

**Figure 11:**
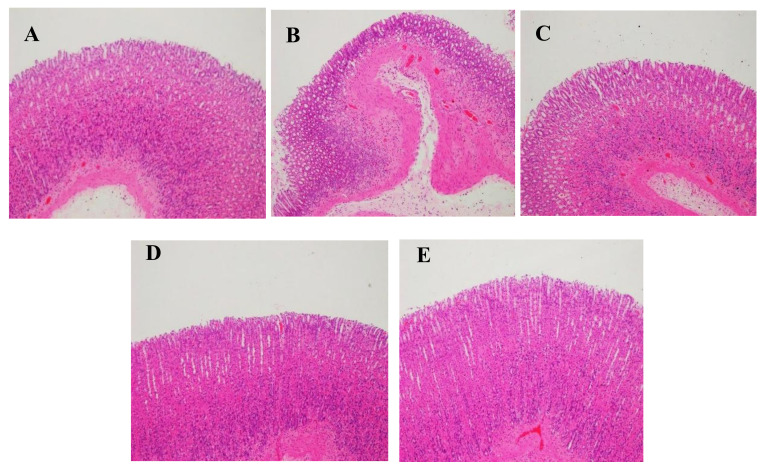
Microscopic appearance of gastric mucosa showing the effect of Zuojin powder and alkaloids′ combination in reflux gastritis rats (HE × 100): (**A**): normal control group; (**B**): model group; (**C**): magnesium aluminum carbonate group; (**D**): Zuojin powder group; (**E**): alkaloids′ combination group.

As seen in the microscopic appearance of gastric mucosa in Figure 11, in the normal control group, the gastric antrum mucosa epithelial cells and glands were well-organized and the boundary between glandular epithelium and glandular ducts was clear. There was no dilation or congestion, no obvious inflammatory cell infiltration, and no obvious abnormality in the mucosal layer. In the model group, the gastric antrum mucosa epithelium was obviously damaged and inflammatory cell infiltration was observed, with moderate or severe inflammation. The gastric antrum mucosa epithelial cells in the Zuojin powder and alkaloids’ combination groups were more integral, with very slight damage and less inflammatory cell infiltration, which was closer to the mucosa of gastric antrum in the normal control group. In the aluminum magnesium carbonate group, the gastric antrum mucosa epithelial cells and glands were well-organized, with a few inflammatory cells infiltrated.

## 3. Materials and Methods

### 3.1. Materials and Reagents

The crude drug of CR was collected from bozhou of China (Batch No. 200528), and that of EF was collected from Hubei of China (Batch No. 1812002). Macroporous resins D101, AB-8, D201, LX-69B, and LSA-10 were collected from Xi′an Lanxiao Co. Ltd. (Xi′an, China). Reference standards of berberine hydrochloride (Lot 110713-202015, purity > 86.8%), palmatine chloride (Lot 110732-201913, purity > 86.8%), evodiamine (Lot 110802-2011710, purity > 99.4%), jatrorrhizine chloride (Lot 110733-201609, purity > 89.5%), and coptisine chloride (Lot 112026-201802, purity > 99.5%) were acquired from National Institutes for Food and Drug Control (Beijing, China). Epiberberine (Lot JOT-10153, purity > 98%), columbamine (Lot 18011702, purity > 91%), rutaecarpine (Lot JOT-10153, purity > 99.5%), and dehydroevodiamine (Lot JOT-11252, purity > 99.8%) were purchased from Chengdu Gelipu Co., Ltd. (Chengdu, China). HPLC-grade acetonitrile (Thermo Fisher Scientific, NY, USA) and ultra-pure water (Hangzhou Wahaha Group Co. LTD) were used in the mobile phase. Methanol and formic acid (AR-grade) for sample preparation were obtained from Tianjin Damao Co. Ltd. (Tianjin, China).

### 3.2. Chromatographic Conditions

Quantitative determination was performed with a Waters ACQUITY UPLC H-Class PLUS system (Waters Corporation, Milford, MA, USA) equipped with a quaternary pump, a column oven, an autosampler, and photodiode array detectors (PDA). Qualitative analysis was performed with an Agilent 1290 Infinity II UHPLC system coupled to an G6530C Q-TOF-MS mass spectrometer equipped with a quaternary pump, a diode-array detector (DAD), a column oven, an autosampler, and an ESI source (Agilent Technologies, Inc., Palo Alto, CA, USA), and data analysis was performed with Agilent Qualitative Navigator (B.08.00) and Qualitative Workflows (B.08.00) software.

All qualitative and quantitative separation was successfully performed with a Waters ACQUITY UPLC^®^ BEH Shield RP18 column (2.1 × 100 mm, 1.7 µm). The mobile phases for the quantitative method were composed of (A) acetonitrile and (B) 0.1% formic acid aqueous solution.

The optimized gradient elution was as follows: 0–3.6 min, 5–11.5% A; 3.6–10 min,11.5–15% A; 10–20 min, 15–19% A; and 20–30 min, 19–85% A; The flow rate was 0.4 mL·min^−1^, the inject volume was 1 µL, and the column temperature was set at 30 °C. The detection wavelength was set at 270 nm.

During qualitative analysis, mass spectrometry detection was performed in both positive and negative ion modes. The drying temperature was 350 °C, the drying gas flow rate was 10 L·min^−1^, the pressure of nebulizer gas was 35 psi, the sheath gas temperature was 350 °C, the sheath gas flow rate was 12 L·min^−1^, and the capillary voltages were 4000 V (positive mode) and 3500 V (negative mode). The MS mode was selected for primary mass spectrometry with a quality scanning range of *m/z* 100–1300. The auto-MS/MS mode was used for the secondary mass spectrum, and the collision voltages were 10, 20, and 30 V. The optimized gradient elution for the purified TAs of EF was as follows: 0–3.6 min, 5–11.5% A; 3.6–10 min, 11.5–15% A; 10–20 min, 15–19% A; and 20–40 min, 19–85% A. The same method was conducted with the purified TAs of CR for qualitative determination. LC–MS data were collected with Agilent MassHunter (B.08.00) software.

### 3.3. Preparation of Sample and Standard Solutions for UHPLC

#### 3.3.1. Preparation of Standard Solutions

The reference standards of dehydroevodiamine, coptisine chloride, epiberberine, columbamine, jatrorrhizine, berberine hydrochloride, palmatine chloride, evodiamine, and rutaecarpine were dissolved with MeOH and stocked at 4 °C. The reference compound stock solutions of berberine, coptisine, epiberberine, palmatine, jatrorrhizine, and columbamine were mixed to obtain the mixed reference standard solution A. The final concentrations of those solutions in A were 187.8, 89.3, 51.0, 84.7, 43.0, and 46.8 µg·mL^−1^, respectively. Dehydroevodiamine, evodiamine, and rutaecarpine were mixed to obtain standard solution B. The final concentrations of those solutions in B were 47.3, 31.5, and 20.4 µg·mL^−1^, respectively. 

#### 3.3.2. Preparation of Sample Solutions

The crude drugs of CR and EF: CR and ER decoction pieces were ground into powder with a particle mesh size of 50 before use. An amount of 0.1 g CR and EF herbal powder was accurately weighed and soaked in 25 mL of an MeOH–HCl solution (100:1; *v/v*) for 1 h and then ultrasonic-extracted for 30 min. The weight loss in the ultrasonic extraction procedure was compensated, and the extracted solution was centrifuged at 13,000 r/min for 10 min; then, the supernatant was removed for analysis.

Purified TAs of CR and EF: purified TA powder (0.1 g) was accurately weighed and soaked in 25 mL of MeOH and then ultrasonic extracted for 30 min. The supernatant was removed for analysis.

### 3.4. UHPLC–DAD Method Validation

The linearity, precision, repeatability, stability, and accuracy were evaluated to validate the proposed UHPLC–DAD method. Solutions were injected for detection at an appropriate volume, and the calibration curves were established by determining the peak areas against the compound amount (µg) of each analyte. The intra- and inter-day precision was validated with mixed solutions under the optimized conditions six times in the same day and once a day for three consecutive days, respectively. The repeatability was performed by analyzing six independently prepared sample solutions. The stability was carried out with one sample at solution intervals of 0, 2, 4, 8, 12, and 36 h. For the recovery test, accurate amounts of the nine alkaloid standard solutions were spiked into appropriate volumes and analyzed. 

### 3.5. Determination of the TAs Content by Ultraviolet Spectrophotometry

The content of TA was determined using the Folin–Ciocalteu colorimetric method (FC method) with slight modifications, and the TA content was calculated as berberine (CR) and evodiamine (EF) equivalents using dynamic calibration curves.

### 3.6. Static Adsorption and Desorption Test 

#### 3.6.1. Extraction 

The CR or EF crude drug (400 g) was refluxed with 70% (*v/v*) ethanol (2 × 4 L, 1 h), and the extract solution was filtered and then concentrated the solvent to a concentration of 0.5 g·mL^−1^ (calculated as the weight of crude drug). 

#### 3.6.2. Resin Selection 

Macroporous resins D101, D201, AB-8, LX-69B, and LSA-10 (3 g) were precisely weighed and separately placed in 100 mL conical flasks, 30 mL of CR or EF were added, and the mixtures were stirred and shaken for 12 h. The absorbance of the supernatant was determined with UV spectrophotometry, and the adsorption capacity of the resin was expressed as TA content (mg TAs·g^−1^ dry resin). After the resin was dried with filter paper, 30 mL of 95% ethanol were precisely added, stirred, and shaken for 12 h. The supernatant was carefully diluted to determine the absorbance. The desorption rate was calculated with the adsorption and desorption capacities. 

#### 3.6.3. Adsorption and Desorption Time 

The D101 macroporous resin (3 g) was accurately weighed and placed in 100 mL conical flasks; then, 30 mL of CR or EF were added, stirred, and shaken. At 20, 40, 60, 120, 180, 240, 300, and 360 min, the absorbance of supernatant was determined with UV spectrophotometry, and the adsorption capacity was calculated as total alkaloids. Then, filter paper was used to dry the surface of the D101 resin, and 30 mL of 95% ethanol were precisely added to desorb. At 20, 40, 60, 120, 180, 240, 300, and 360 min, the supernatant was diluted, and the absorbance of the supernatant was determined with UV spectrophotometry. 

#### 3.6.4. pH 

The pH of CR or EF (9 × 30 mL) was adjusted to 2, 3, 4, 5, 6, 7, 8, 9, and 10. The samples were added to the D101 macroporous resin and adsorbed for 6 h, and the TA content was determined. 

#### 3.6.5. Temperature 

The 70% ethanol extracts (4 × 30 mL) of CR or EF were adjusted to 25, 35, 45, and 55 °C before added to the D101 macroporous resin. Then, they were adsorbed for 6 h, and the TA content was determined. 

### 3.7. Dynamics Adsorption and Desorption

The preprocessed D101 resins were wet-loaded into the glass column (D = 2 cm; H = 16 cm) to carry out the dynamics adsorption and desorption experiments. Firstly, 25 mL of CR and EF were flowed through the resin columns at a speed of 1 BV·h^−1^. After the adsorption equilibrium, ultrapure water (5 BV) was used to wash the resin columns to remove impurities such as sugar and pigments. Finally, 8 different BV concentrations of ethanol (20%, 40%, 60%, 80%, and 95% *v/v*) were used to desorb the alkaloids or limonin, and the fractions (0.5 BV) were further concentrated and dried to calculate the amount of TAs.

### 3.8. Sample Preparation of CR and EF Purified TAs

Crude drugs (0.2, 0.4, and 2 kg) of CR or EF were refluxed twice with 70% (*v/v*) ethanol and loaded onto D101 macroporous resin columns by using the optimized procedure. 

### 3.9. Inhibitory Effect of CR and EF Alkaloids’ Combinations in BRG Rats

#### 3.9.1. Materials

Adult male Sprague–Dawley (SD) rats—8 weeks old, weighing 180–200 g, and half male and half female—were purchased from the SPF (Beijing, China) Biotechnology Co., Ltd. (SCXK (Beijing) 2017-0026). They were fed in a specific pathogen-free environment with 12 h of light a day with unlimited drinking water. Taurocholic acid sodium was manufactured by Beijing Biotopped Technology Co., Ltd., Beijing, China (CAS No. S0342). Lecithin was manufactured by Shanghai Macklin Biochemical Co., Ltd., Shanghai, China. (CAS No. 8002-43-5). Pancreatin was manufactured by Gibco Inc., Shanghai, China. (CAS No. 9002-07-7). A kind of liquor (2.5 g of taurocholic acid sodium, 1.5 g of lecithin, and 0.25 g of pancreatin dissolved in 100 mL of water) was used to establish a reflux gastritis model in rats [30,31,32]. Magnesium aluminum carbonate was pursued from Bayer healthcare Co., Ltd., Beijing, China. (Batch No. JS14966). Zuojin powder: EF and CR crude drug powder were mixed at a ratio of 6:1 (Batch No. G-20201218-01). EF and CR alkaloids′ combination: purified TAs of CR and EF were calculated and mixed with a portion of the crude drug in the Zuojin Pill (6:1). As calculated by yield rate, 2.2 g·kg^−1^ Zuojin powder could be converted to 0.24 g·kg^−1^ alkaloids′ combination (Batch No. G-20210122-01).

#### 3.9.2. Animal Treatments

**Figure 12:**
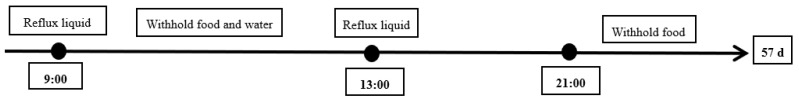
Modeling process of reflux gastritis rats.

The reflux gastritis model was built by following an established procedure [30,31,32]. Reflux liquid was used for feeding the laboratory rats by gastric irrigation. The dose of reflux liquid was 15 mL·kg^−1^ body weight, 2 times a day, with an interval of 4 h. All laboratory rats were administered treatment for 57 d except for 10 rats in the normal control group (Figure 12).

**Figure 13:**
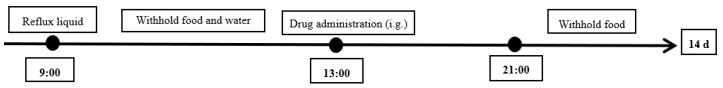
Administration process of reflux gastritis rats.

After the above modeling process, 40 SD rats were randomly divided into 4 groups: model group, magnesium aluminum carbonate group, Zuojin powder group, and alkaloids′ combination group. Magnesium aluminum carbonate, Zuojin powder, and alkaloids′ combination were dissolved or suspended in water, and the rats were administrated 15 mL·kg^−1^ body weight, once a day, for 14 d (Figure 13).

Animal welfare and experimental procedures were carried out strictly in accordance with the Guide for the Care and Use of Laboratory Animals (National Institutes of Health, Bethesda, MD, USA) and the related ethical regulations of animal care and use committee of Increasepharm Safety and Efficacy Co., Ltd. (Beijing, China). All efforts were made to minimize animals’ suffering and to reduce the number of animals used.

#### 3.9.3. Specimen Collection

The animals were killed after administration 14 d into the experiment. The abdomen was immediately opened. The whole stomach was cut and fixed in 10% formalin solution, gastric mucosa tissue was taken for HE staining, the pathological degree of gastric mucosa was observed under microscope, and the differences between groups were compared according to semi-quantitative grading standards. 

#### 3.9.4. Determination of the Pathological Degree of Gastric Mucosa with Microscope 

After the gastric mucosa tissue was fixed, the gastric antrum tissue of appropriate size was cut out for paraffin embedding, sectioning, and HE staining. The infiltration of inflammatory cells in gastric antrum mucosa and metaplasia of intestinal epithelial cells were observed with semi-quantitative detection under microscope. At magnification (100×), 5 fields of each pathological section were randomly selected for inflammatory scoring. Normal (−) signifies no more than 5 mononuclear cells per magnification field and no obvious intestinal metaplasia in gastric mucosa. Mild (+) signifies few chronic inflammatory cells or metaplasia of glandular intestinal epithelium, accounting for no more than 1/3 of the mucosal layer. Moderate (++) signifies more chronic inflammatory cells or metaplasia of glandular intestinal epithelium than the normal, accounting for 1/3 to 2/3 of the mucosal layer. Severe (+++) signifies more chronic inflammatory cells or metaplasia of glandular intestinal epithelium than the mild, accounting for the whole mucosa layer. Lymphatic follicles were not included in the calculation. An intuitive analog scoring method was used to level, and the levels were 0, 1, 2, and 3 corresponding to −, +, ++, and +++, respectively [33,34] (Table 6).

### 3.10. Statistical Analysis

All experiments were performed in triplicate (*n* = 3). The results were expressed as the means ± standard deviation. The experiment data were statistically analyzed with Microsoft Excel. In all statistical analyses, *p* values of ≤0.05 were regarded as statistically significant and *p* values ≤ 0.01 were regarded as very significant.

## 4. Conclusions

In conclusion, the authors of this study developed a simple and efficient method for the preliminary separation and purification of alkaloids from CR and EF herbal extracts. Among the five investigated typical macroporous resins, the D101 resin was selected due to its best adsorption capacity, desorption capacity, and ratio of desorption. Optimized adsorption parameters were selected. Further dynamic adsorption/desorption experiments on a D101 column were also conducted to obtain the optimal parameters. Crude drugs of CR and EF were extracted and purified according to the optimized purification process, respectively. Then, a UHPLC–ESI–QTOF-MS method for qualitative analysis was developed. Thirty-one compounds—thirty alkaloids and one phenolic compound—were identified or tentatively assigned by comparison with reference standards or literature data. The contents of nine index components in crude drugs and purified TAs were determined with the UHPLC–DAD method. Six alkaloids in the CR crude drug—coptisine, epiberberine, columbamine, jatrorrhizine, palmatine, and berberine—were found to account for 1.5%, 0.89%, 0.47%, 0.35%, 1.19%, and 4.83%, respectively. In the purified TAs of CR, the contents of the six alkaloids were 9.77%, 5.41%, 3.14%, 2.24%, 7.96%, and 31.46%, respectively. In addition to CR, the contents of three major marker alkaloids in the purified TAs of EF were also significantly increased: the contents of evodiamine, rutaecarpine, and dehydroevodiamine in the EF crude drug were 0.55%, 0.28%, and 0.86%, respectively. After purification, the contents were 5.89%, 5.07%, and 0.92% respectively. Limonin was not detected in the purified TAs of EF. The results verified a good adsorption and desorption chromatography for the preparative enrichment and separation of alkaloids from medical herbs. Moreover, the purified TAs of CR and EF were calculated and mixed with a portion of crude drug in the Zuojin Pill (6:1), and the alkaloids′ combination could markedly attenuate the pathological changes of gastric mucosa in rats. It is expected that this research will provide comprehensive analysis information for the enrichment and purification process, which can prepare high purity total alkaloids from ethanol extracts of CR and EF, and furthermore provide a material basis for the pharmacological study of the Zuojin Pill in the future.

## Figures and Tables

**Table 1:** Physical properties of five macroporous resins.

Resins	Structure	Surface Area (m^2^/g)	Average Pore Diameter (Å)	Polarity	Moisture Content (%)
D101	Polystyrene	400–550	200–300	No-polar	65.61
D201	Polystyrene	≥650	150–200	Strong-base anion	49.43
AB-8	Polystyrene	450–520	130–140	Weak-polar	64.46
LX-69B	Polystyrene, divinylbenzene	≥1000	120–140	Polar	59.60
LSA-10	Polystyrene, divinylbenzene	≥500	210–260	Polar	60.70

**Table 2:** Characterization of compounds in purified TAs of CR determined with UHPLC–ESI–QTOF-MS/MS.

No.	Identification	t_R_ (min)	Ion Mode	Mass (*m/z*)	Formula	Fragment Ions (*m/z*)
1	3-(3’,4’-Dihydroxyl)-(2*R*) lactate-4’-oxygen-β-D-glucoside	2.387	[M + NH_4_]^+^	378.1424	C_15_H_20_O_10_	378.1405, 359.1093, 197.0461
2	Magnoflorine	4.896	[M]^+^	342.1706	C_20_H_24_NO_4_^+^	342.1725, 297.1130, 282.0898
3	8-Oxepiberberine	7.916	[M + H]^+^	352.1190	C_20_H_17_NO_5_	352.1186, 336.0871, 322.0718, 308.0924, 294.0758
4	Berberrubine	8.286	[M]^+^	322.1082	C_19_H_16_NO_4_^+^	322.1074, 307.0841, 294.0772, 279.0885
5	2-Hydroxyljatror-rhizine	8.722	[M]^+^	324.1242	C_19_H_18_NO_4_^+^	324.1231, 308.0914, 294.0761, 280.0963, 266.0805
6	Coptisine	9.861	[M]^+^	320.0918	C_19_H_14_NO_4_^+^	320.0932, 292.0976, 277.0746, 262.0864
7	Epiberberine	10.496	[M]^+^	336.1232	C_20_H_18_NO_4_^+^	336.1238, 320.0925, 292.0974
8	Columbamine	11.136	[M]^+^	338.1388	C_20_H_20_NO_4_^+^	338.1392, 322.1086, 308.0929, 294.1135
9	Jatrorrhizine	11.502	[M]^+^	338.1394	C_20_H_20_NO_4_^+^	338.1395, 322.1083, 308.0924, 294.1130, 280.0964
10	Worenine	12.241	[M]^+^	334.1088	C_20_H_16_NO_4_^+^	334.1088, 319.0726
11	Groenlandicine	12.943	[M]^+^	322.1088	C_19_H_16_NO_4_^+^	322.1082, 307.0848, 279.0893
12	Berberine	13.512	[M]^+^	336.1239	C_20_H_18_NO_4_^+^	336.1241, 320.0924, 306.0773, 292.0974, 278.0818
13	Palmatine	12.056	[M]^+^	352.1544	C_21_H_22_NO_4_^+^	352.1550, 336.1235, 322.1080, 294.1125, 278.0814, 264.1018

**Table 3:** Characterization of compounds in purified TAs of EF determined with UHPLC–ESI–QTOF-MS/MS.

No.	Identification	t_R_ (min)	Ion Mode	Mass (*m/z*)	Formula	Fragment ions (*m/z*)
1	Dehydroevodiamine	9.324	[M]^+^	302.1300	C_19_H_16_N_3_O^+^	302.1290, 287.1046, 286.0978, 272.0819, 258.1024
2	2-Hydroxy-4-methoxy-3-(3’-methyl-2’-butenyl)-quinolone	27.292	[M + H]^+^	244.1903	C_15_H_17_NO_2_	244.1903, 228.1016, 200.0703, 186.0904, 173.0839
3	7β-Hydroxyl rutaecarpine	28.734	[M + H]^+^	304.1083	C_18_H_13_N_3_O_2_	304.1083, 286.0971
4	1-Methyl-2-[7-hydroxyl (-*E*)-9-undecenyl]-4(1H)-quinolone	29.839	[M + H]^+^	328.2685	C_21_H_29_NO_2_	328.2685, 310.2171, 186.0910, 173.0833
5	1-Methyl-2-[7-hydroxyl (-*E*)-9-tridenyl]-4(1H)-quinolone	30.408	[M + H]^+^	356.2585	C_23_H_33_NO_2_	356.2585, 338.2472, 186.0920, 173.0822,
6	1-Methyl-2-[7-carbonyl (-*E*)-9-tridecenyl]-4(1H)-quinolone	30.778	[M + H]^+^	354.1435	C_23_H_31_NO_2_	354.1435, 288.1132, 228.1375, 200.1070, 186.0911, 173.0831
7	Evodiamine	30.944	[M + H]^+^	304.1435	C_19_H_17_N_3_O	304.1427, 171.0902, 134.0590
8	Rutaecarpine	31.783	[M + H]^+^	288.1127	C_18_H_13_N_3_O	288.1143, 273.0905, 244.0887, 169.0762
9	1-Methyl-2-nonyl-4(1H)-quinolone	32.419	[M + H]^+^	286.2159	C_19_H_27_NO	286.2159, 242.1545, 214.1218, 200.1066, 186.0913, 173.0834
10	1-Methyl-2-[(Z)-6-undecenyl]-4(1H)-quinolone	33.324	[M + H]^+^	312.2324	C_21_H_29_NO	312.2324, 186.0920, 173.0827
11	1-Methyl-2-[(4Z,7Z)-tridecadienyl]-4(1H)-quinolone	34.533	[M + H]^+^	338.2483	C_23_H_31_NO	338.2481, 212.1065, 186.0909, 173.0831, 159.0675
12	1-Methyl-2-undecyl-4(1H)-quinolone	35.036	[M + H]^+^	314.2486	C_21_H_31_NO	314.2486, 242.1539, 228.1383, 200.1070, 186.0919, 173.0839
13	1-Methyl-2-[(6Z, 9Z, 12E)-pentadeca triene]-4(1H)-quinolone	35.256	[M + H]^+^	364.2638	C_25_H_33_NO	364.2640, 308.2008, 268.1688, 228.1375, 200.1064, 186.0909, 173.0830, 159.0671
14	2-Tridecyl-4 (1H)-quinolone	35.472	[M + H]^+^	328.2668	C_22_H_33_NO	328.2668, 186.0912, 173.0838
15	Evocarpine	35.705	[M + H]^+^	340.2650	C_23_H_33_NO	340.2650, 256.1704, 242.1547
16	1-Methyl-2-[(6Z,9Z)-pentadecadienyl]-4(1H)-quinolone	36.511	[M + H]^+^	366.2782	C_25_H_35_NO	366.2782, 268.1691, 228.1378, 186.0910, 173.0833, 159.0670
17	Dihydroevocarpine	37.550	[M + H]^+^	342.2811	C_23_H_35_NO	342.2791, 200.1066, 186.0913, 173.0833, 159.0668
18	1-Methyl-2-pentadecyl-4(1H)-quinolone	39.830	[M + H]^+^	370.3099	C_25_H_39_NO	370.3110, 200.1069, 186.0918, 173.0838, 159.0676

**Table 4:** The regression equations of nine analytes.

Analytes	Calibration Curve	R^2^	Linear Range (µg·mL*^−^*^1^)
Coptisine	Y = 9249.2x − 10946	0.9995	7.144–178.600
Epiberberine	Y = 9504x − 9426.4	0.9997	4.081–102.034
Columbamine	Y = 8930.2x − 7565.7	0.9998	3.741–93.582
Jatrorrhizine	Y = 10752x − 6106.8	0.9999	3.433–85.831
Berberine	Y = 10509x − 33478	0.9997	15.290–382.255
Palmatine	Y = 11165x − 14955	0.9997	6.774–169.343
Dehydroevodiamine	Y = 1522.8x − 226.55	0.9999	2.959–94.672
Evodiamine	Y = 5370.8x − 132.5	1.0000	1.967–62.947
Rutaecarpine	Y = 3394.9x + 1086.6	1.0000	1.592–50.960

**Table 5:** Precision, stability, repeatability, and accuracy of nine analytes.

Analytes	Precision		Repeatability		Stability	Recovery	
	Intra-Day RSD% (*n* = 6)	Inter-Day RSD% (*n* = 3)	Mean Concentration (%)	RSD% (*n* = 6)	RSD% (*n* = 6)	Average Recovery (%)	RSD% (*n* = 9)
Coptisine	0.87	1.16	7.88	1.28	1.51	99.43	0.77
Epiberberine	0.84	1.14	3.85	1.37	0.90	97.42	1.04
Columbamine	0.88	1.07	2.50	1.81	1.55	100.60	2.53
Jatrorrhizine	0.84	1.05	1.63	1.80	2.42	99.26	2.06
Berberine	1.04	1.13	30.20	1.23	1.08	99.10	2.68
Palmatine	1.18	1.21	6.61	1.18	2.53	98.52	1.56
Dehydroevodiamine	1.05	1.24	1.59	0.88	1.15	99.85	2.42
Evodiamine	0.72	1.19	9.23	0.91	2.30	97.70	2.76
Rutaecarpine	1.11	1.15	5.14	1.40	2.58	100.80	2.93

**Table 6:** Pathological degree of gastric mucosa with microscope.

Levels	Pathological Degree
0	No obvious inflammatory cell infiltration in gastric mucosa and intestinal metaplasia in gastric mucosa
1	Very little infiltration of chronic inflammatory cells or metaplasia of glandular intestinal epithelium, involving only the 1/3 mucosal layer
2	Very little infiltration of chronic inflammatory cells or metaplasia of glandular intestinal epithelium, involving the 2/3 mucosal layer
3	More Chronic inflammatory cells infiltration or glandular intestinal metaplasia, involving the whole layer of mucosa

## Data Availability

The data presented in this study are available on request from the corresponding authors.

## Sample Availability

Samples are available from the authors.
